# Genomic surveillance of *Escherichia coli* in municipal wastewater treatment plants as an indicator of clinically relevant pathogens and their resistance genes

**DOI:** 10.1099/mgen.0.000267

**Published:** 2019-05-20

**Authors:** Kathy E. Raven, Catherine Ludden, Theodore Gouliouris, Beth Blane, Plamena Naydenova, Nicholas M. Brown, Julian Parkhill, Sharon J. Peacock

**Affiliations:** 1 Department of Medicine, University of Cambridge, Box 157 Addenbrooke’s Hospital, Hills Road, Cambridge CB2 0QQ, UK; 2 London School of Hygiene and Tropical Medicine, Keppel Street, London WC1E 7HT, UK; 3 Clinical Microbiology and Public Health Laboratory, Public Health England, Cambridge CB2 0QQ, UK; 4 Wellcome Sanger Institute, Wellcome Trust Genome Campus, Hinxton, Cambridge CB10 1SA, UK

**Keywords:** *Escherichia coli*, genomic surveillance, resistance, wastewater

## Abstract

We examined whether genomic surveillance of *Escherichia coli* in wastewater could capture the dominant *E. coli* lineages associated with bloodstream infection and livestock in the East of England, together with the antibiotic-resistance genes circulating in the wider *E. coli* population. Treated and untreated wastewater was taken from 20 municipal treatment plants in the East of England, half in direct receipt of acute hospital waste. All samples were culture positive for *E. coli*, and all but one were positive for extended-spectrum β-lactamase (ESBL)-producing *E. coli*. The most stringent wastewater treatment (tertiary including UV light) did not eradicate ESBL-*E. coli* in 2/3 cases. We sequenced 388 *E. coli* (192 ESBL, 196 non-ESBL). Multilocus sequence type (ST) diversity was similar between plants in direct receipt of hospital waste versus the remainder (93 vs 95 STs, respectively). We compared the genomes of wastewater *E. coli* with isolates from bloodstream infection (*n*=437), and livestock farms and retail meat (*n*=431) in the East of England. A total of 19/20 wastewater plants contained one or more of the three most common STs associated with bloodstream infection (ST131, ST73, ST95), and 14/20 contained the most common livestock ST (ST10). In an analysis of 1254 genomes (2 cryptic *E. coli* were excluded), wastewater isolates were distributed across the phylogeny and intermixed with isolates from humans and livestock. Ten *bla*_CTX-M_ elements were identified in *E. coli* isolated from wastewater, together with a further 47 genes encoding resistance to the major antibiotic drug groups. Genes encoding resistance to colistin and the carbapenems were not detected. Genomic surveillance of *E. coli* in wastewater could be used to monitor new and circulating lineages and resistance determinants of public-health importance.

## Data Summary

The study sequences are available in the European Nucleotide Archive (https://www.ebi.ac.uk/ena) under the project numbers PRJEB8770 and PRJEB8768. Additional external sequences were downloaded from the European Nucleotide Archive with the accession numbers U00096.2, LT632320.1 and ERR1202273.

**Impact Statement**

*Escherichia coli* is a leading cause of urinary tract and bloodstream infections, the rates of which are increasing. Of particular concern are antibiotic-resistant strains, especially those resistant to the carbapenem drugs and colistin. As rates of antibiotic-resistant *E. coli* infection increase, sustainable mechanisms will be required to monitor new and circulating strains and antibiotic resistance. Municipal sewage plants provide readily accessible sites that contain bacterial populations drawn from across geographically defined regions. We showed that genomic surveillance of *E. coli* in wastewater detected the dominant *E. coli* lineages associated with serious human disease and carriage by livestock in the East of England, together with a snapshot of antibiotic-resistance genes in the wider circulating *E. coli* population. This approach could be used to monitor trends in such characteristics over time.

## Introduction

*Escherichia coli* is a leading cause of urinary tract and bloodstream infections [1], the rates of which are increasing. For example, the incidence of *E. coli* bacteraemia in England has increased year-on-year, from 32 405 cases in 2012 to 40 272 cases in 2016, an overall increase over the 5 year period of 24.3 % [2]. Around 75 % of *E. coli* bacteraemias have a community onset, indicating both community and hospital reservoirs of invasive *E. coli*. Of particular concern are the rising rates of infection with antibiotic-resistant *E. coli,* especially those producing extended-spectrum β-lactamases (ESBLs) that hydrolyse third-generation cephalosporins, and *E. coli* with acquired resistance to the carbapenems and colistin. *E. coli* infections caused by these antibiotic-resistant strains have an adverse impact on patient outcome and length of hospital stay compared to infection with antibiotic-susceptible *E. coli* [1, 3, 4].

As rates of antibiotic-resistant *E. coli* infection increase, sustainable mechanisms will be required to monitor new and circulating lineages and their resistance determinants. Municipal sewage plants provide readily accessible sites that contain bacterial populations drawn from geographically defined regions. It has been proposed that surveillance of antibiotic-resistant lineages, resistance genes and mobile elements in untreated sewage could provide a surrogate indicator for regional antimicrobial resistance and how this changes over time [5, 6]. Sewage could be sampled from hospitals to survey inpatient populations, and from wastewater treatment plants that serve urban and rural populations. Previous studies have demonstrated that treated wastewater released into surface waters contains drug-resistant pathogens [7, 8], and sampling wastewater treatment outflows could monitor the burden of drug-resistant pathogens released into surface waters.

The application of sequencing technologies to wastewater has great potential for the detection of pathogenic clones and circulating antibiotic-resistance genes, although its application to this reservoir has been relatively limited to date. Using metagenomic analyses and 16S rRNA sequencing, extensive sharing of antibiotic-resistance genes and bacterial communities has been observed between wastewater treatment plants in China [9]. Studies using whole-genome sequencing confirmed the presence of the same ESBL *E. coli* multilocus sequences types (STs) in recreational waters, wastewater from a nearby plant and from human urinary samples in Norway [10], and a study from Thailand identified genetically related strains from clinical and canal water isolate collections that were located in close geographical proximity [11]. The aim of this study was to extend our current understanding of the utility of genomic surveillance of sewage by evaluating whether the sequencing of *E. coli* isolated from multiple municipal wastewater treatment plants could support the surveillance of pathogenic *E. coli* lineages and resistance genes in a defined region of the UK.

## Methods

### Wastewater sampling and microbiology

A cross-sectional survey was conducted between June 2014 and January 2015 to isolate *E. coli* from raw and treated wastewater collected from 20 municipal wastewater treatment plants across the East of England. Of these, 10 were located downstream of acute hospitals and 10 did not directly receive hospital waste. The treatment type was recorded for each plant, as follows: secondary treatment [this follows preliminary and primary treatment and involves biological treatment processes (bacterial breakdown) by an activated sludge process or filter beds]; and tertiary treatment [such as lagooning (reed beds) or sand filtration]. We noted whether tertiary treatment included disinfection by UV light, which is used to reduce pathogenic bacterial and viral organisms in treated water that is discharged into sensitive areas such as freshwater, estuarine and coastal locations [12].

Treated and untreated wastewater samples were obtained from each plant. At each sampling point, two consecutive grab samples of 0.5 l each were collected and placed into 1 l sterile bottles containing 18 mg sodium thiosulphate (Sigma-Aldrich). Triplicate serial dilutions of 1 ml (to 10^−3^ for treated and to 10^−5^ for untreated wastewater) together with 1, 10 and 100 ml (the latter for treated wastewater only) of neat sample were concentrated using membrane filtration. The resulting membranes were cultured for 48 h at 37 °C in air on the surface of Chromocult agar (VWR) to detect and enumerate *E. coli* and on *Brilliance* ESBL agar (Oxoid) to select and enumerate ESBL *E. coli*. At least one colony of each morphology type suspected to be *E. coli* was selected and the species confirmed using MALDI-TOF MS (Biotyper version 3.1; Bruker Daltonics). Colony forming units (c.f.u.) of *E. coli* and ESBL *E. coli* were counted on plates containing 10–80 colonies, and log reductions between pre- and post-treated water calculated based on c.f.u. ml^−^^1^+1.

Antimicrobial susceptibility was determined using a Vitek 2 instrument (bioMérieux) with an N206 card. Two isolates with the *bla*_OXA-335_ gene were further tested for phenotypic carbapenem resistance using the Rosco test (Rosco Diagnostica). Up to five colonies of ESBL *E. coli* and five colonies of non-ESBL *E. coli* from each untreated and treated wastewater sample were sequenced, resulting in a total of 199 non-ESBL *E. coli* and 195 ESBL *E. coli*. Three non-ESBL *E. coli* and three ESBL *E. coli* isolates were subsequently removed from further analysis after failing sequencing quality-control checks.

### Sequencing and bioinformatic analyses

Genomic DNA was extracted from *E. coli* using a QIAxtractor (Qiagen), according to the manufacturer's instructions. Library preparation was conducted according to the Illumina protocol and sequenced on a HiSeq2000 (Illumina) at the Wellcome Sanger Institute, UK. STs were identified using the MLST sequence archive (https://enterobase.warwick.ac.uk). Assemblies were created using Velvet with the improvements available at https://github.com/sanger-pathogens/vr-codebase and https://github.com/sanger-pathogens/assembly_improvement.

The 388 wastewater isolate genomes were combined with genomes from the following sources: 437 *E. coli* from blood cultures from patients at the Cambridge University Hospitals NHS Foundation Trust (13 *E. coli* sequenced during this study that were isolated between 2014 and 2015, and 424 *E. coli* that were isolated between 2006 and 2013 and reported previously, for which the genomes were publicly available [13]); 411 isolates from 29 livestock farms (cattle, pig, chicken and turkey) in the East of England from 2014 and 2015 [14]; and 20 isolates cultured from retail meat from supermarkets in Cambridge, UK, from 2015 [14] (Table S1, available in the online version of this article). The pangenome was estimated using Roary with default parameters and percentage identity set to 90 %, and SNPs in the core genes were identified [15]. Isolates belonging to three sequence types (STs) (ST10, ST131 and ST117) were each mapped against an ST-specific reference [MG1655 K-12 (accession number U00096.2) for ST10, NCTC13441 (accession number LT632320.1) for ST131 and a *de novo* assembly of a study isolate with accession number ERR1202273 for ST117] using SMALT (http://www.sanger.ac.uk/resources/software/smalt/). Mobile genetic elements were identified and removed as described previously [16], and recombination was removed using Gubbins. SNPs were identified [15] and phylogenies created using RAxML with 100 bootstraps and a midpoint root. Identification of antimicrobial-resistance genes associated with all the major drug groups was performed with ARIBA using the ResFinder database. Mobile genetic elements encoding *bla*_CTX-M-1_, *bla*_CTX-M-15_, *bla*_CTX-M-14_, *bla*_CTX-M-14b_ and *bla*_CTX-M-27_ were identified as previously described [14].

## Results

### Effect of wastewater treatment on bacterial counts

**Fig. 1.**
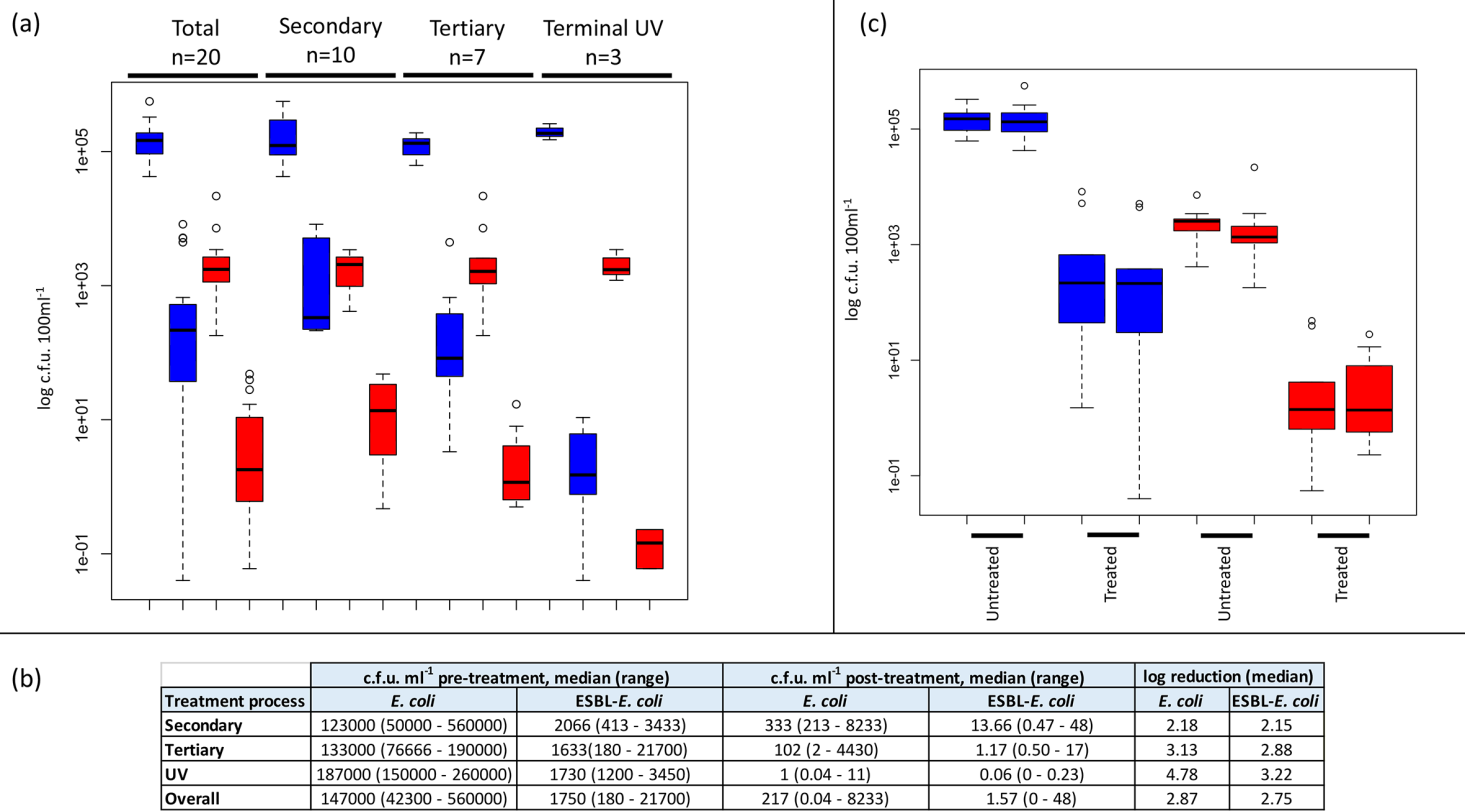
Bacterial counts based on wastewater treatment and relationship to acute NHS hospitals. (a) Boxplot showing bacterial counts in wastewater from treatment plants based on the highest level of treatment used at the plant. Blue, *E. coli*; red, ESBL *E. coli*. For each pair of coloured blocks, the left shows untreated wastewater and the right treated wastewater. The highest level of treatment at the plant is shown at the top of the plot. (b) Table showing the number of c.f.u. in untreated wastewater and in treated water at the point of release into the environment, and the reduction in bacterial counts between the two by treatment type. (c) Boxplot showing bacterial counts in untreated and treated wastewater at plants that were (left block for each pair) or were not (right block) in direct receipt of waste from acute hospitals. Blue, *E. coli*; red, ESBL *E. coli*.

We performed a cross-sectional survey of 20 municipal wastewater treatment plants across the East of England between 2014 and 2015, 10 of which were in direct receipt of waste from acute NHS hospitals (Fig. S1). The highest level of treatment processes used at each plant were secondary (*n*=7), tertiary (*n*=10) or terminal UV light disinfection (*n*=3) (see Table S2 for further details). *E. coli* was isolated from untreated and treated water samples from all 20 plants. Using selective culture medium, ESBL *E. coli* was isolated from all 20 untreated and 19/20 treated samples, the exception being a sample that had been treated with UV light (Table S2). All treatment types led to a reduction in *E. coli* count between untreated and treated water, with a median log reduction of 2.87 for all *E. coli* (grown in the absence of antibiotic selection) and 2.75 for ESBL *E. coli* (Fig. 1a, b). The mean count of *E. coli* and ESBL *E. coli* released into the environment in treated water was 217 and 2 c.f.u. ml^−^^1^, respectively (Fig. 1b). The c.f.u. count was lowest in water treated with UV light (Fig. 1b). The bacterial count was compared for plants in direct receipt of hospital waste versus plants that were not. Counts were not significantly different between the two groups for untreated water (*E. coliP*=0.72, ESBL *E. coliP*=0.64), or for treated water (*E. coliP*=0.72, ESBL *E. coliP*=0.67) (Fig. 1c).

### Genetics of wastewater *E. coli*

**Fig. 2.**
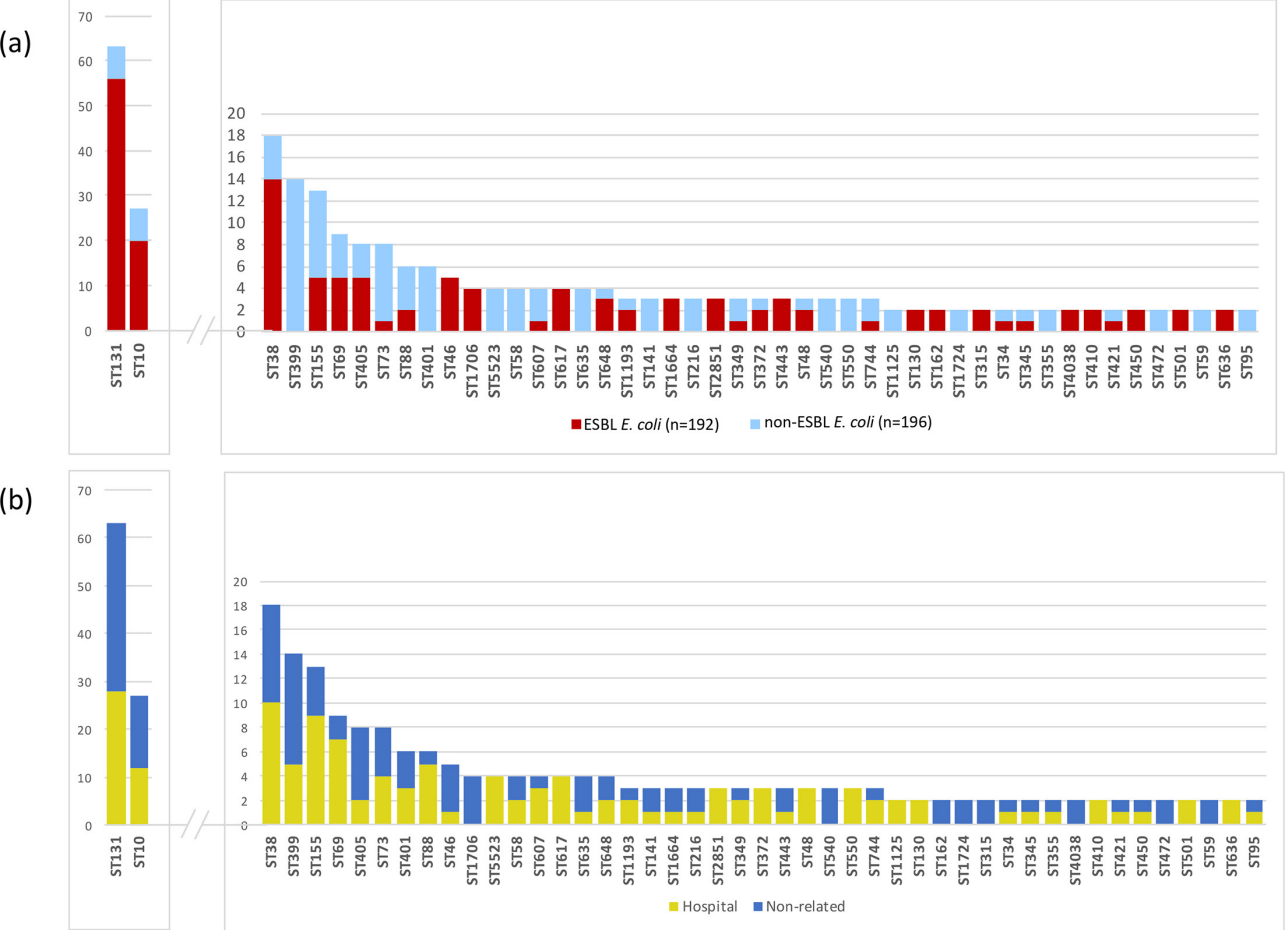
Distribution of STs for non-ESBL and ESBL *E. coli* (a), and between wastewater treatment plants based on direct receipt of hospital waste (b). Graphs show the total number of isolates in each category for all wastewater isolates (*n*=388).

**Fig. 3.**
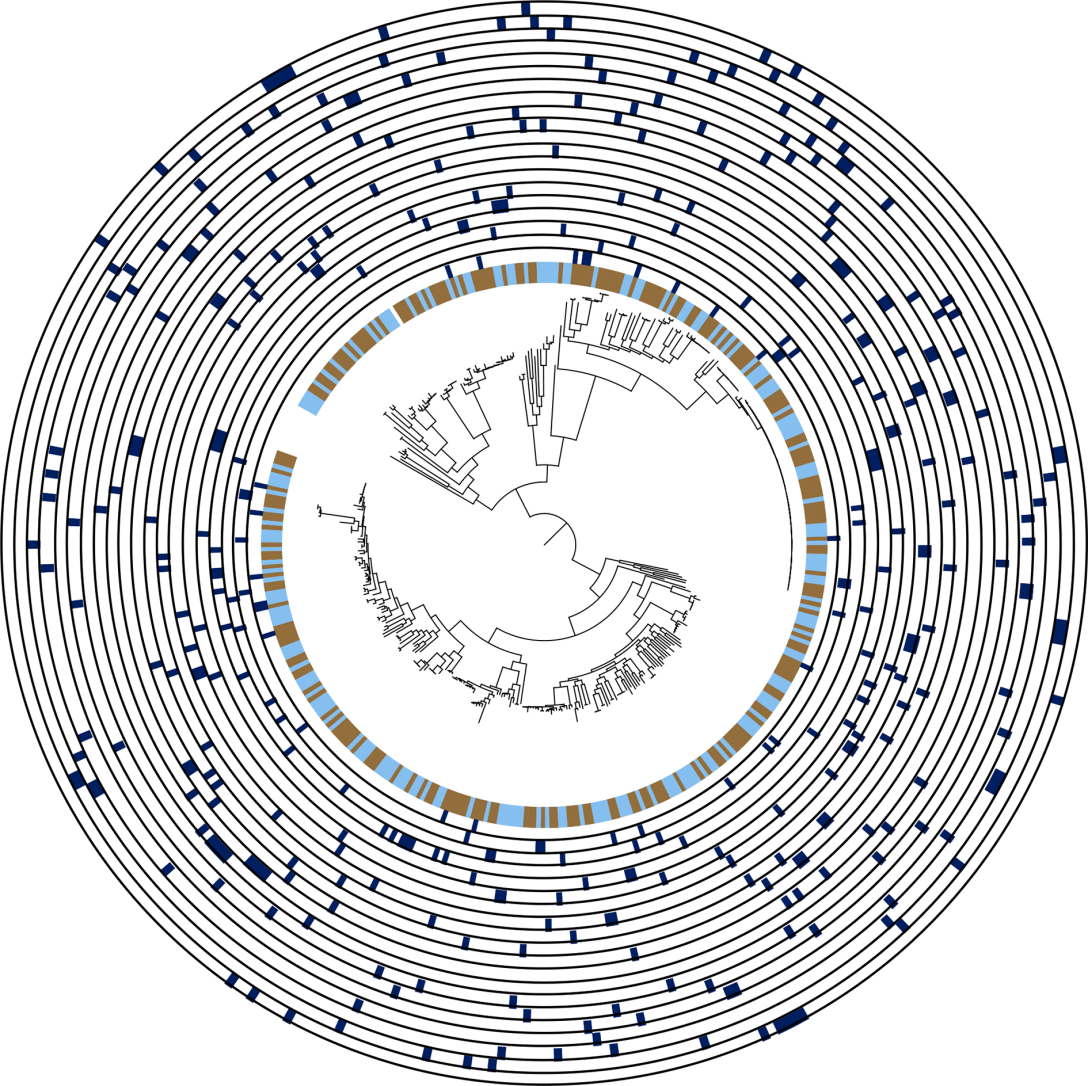
Diversity of *E. coli* in wastewater treatment plants. Maximum-likelihood phylogenetic tree based on SNPs in the core genes for the wastewater collection excluding two cryptic *E. coli* isolates (*n*=386). Inner ring shows whether the isolate was from treated (blue) or untreated (brown) wastewater. Further rings show isolates belonging to each wastewater treatment plant (dark blue), from VREW0001 (innermost ring) to VREW0020 (outermost ring).

A total of 388 wastewater *E. coli* isolates (196 non-ESBL, 192 ESBL) were sequenced, consisting of up to five colonies each of non-ESBL and ESBL *E. coli* from every untreated and treated water sample. MLST analysis identified 160 different STs, with greater diversity for non-ESBL *E. coli* than ESBL *E. coli* (116 versus 64 different STs). The most frequent non-ESBL *E. coli* STs were ST399 (*n*=14, 7 %), ST155 (*n*=8, 4 %) and ST73 (*n*=7, 4 %), and the most frequent ESBL *E. coli* STs were ST131 (*n*=56, 29 %), ST10 (*n*=20, 10 %) and ST38 (*n*=14, 7 %) (Fig. 2a). The diversity of STs was similar between untreated and treated wastewater (101 vs 92 STs, respectively), and between plants that were in direct receipt of hospital waste versus those that were not (93 vs 95 STs, respectively). Plants in direct receipt of hospital waste and those that were not both contained the most common STs, with any variation between the two limited to low-frequency STs (Fig. 2b). A maximum-likelihood phylogenetic tree based on SNPs in the core genes identified two outliers that belonged to cryptic clades III and V (defined as ST2379 and ST133, respectively), which were removed from further analysis. Genetic diversity of *E. coli* isolated from each individual plant was high, with isolates from a given plant distributed across the phylogeny (Fig. 3). There was evidence for highly related isolates between plants, with 8 isolate pairs less than 10 SNPs apart on pairwise core genome comparison (ST131, *n*=4; ST443, *n*=3; ST4028 *n*=1; Fig. S2).

### Comparison of isolates from wastewater, livestock and clinical infection

To investigate the relatedness between isolates from wastewater and those from humans and livestock, we combined the 386 wastewater *E. coli* genomes generated during this study with 437 bacterial genomes associated with bloodstream infection in patients admitted to Cambridge University Hospitals NHS Foundation Trust, 411 isolates from livestock farms and 20 isolates cultured from retail meat, all from the East of England. Place of onset for the bloodstream infections was defined as hospital-associated for 122 patients, community-associated for 286 patients and unknown for the remaining 29 patients. Comparison of STs revealed that 19/20 wastewater plants contained one or more of the three most common STs identified from blood cultures (ST131, ST73 and ST95), the exception being an urban wastewater plant that was not downstream of a hospital. ST131 was assigned to 17 % of blood culture isolates (28 ESBL, 48 non-ESBL), and 16 % of wastewater isolates (56 ESBL, 7 non-ESBL) from 18/20 plants, the 2 exceptions being plants not downstream of hospitals. ST73 was assigned to 16 % of blood culture isolates (9 ESBL, 60 non-ESBL) but only 2 % wastewater isolates (1 ESBL, 7 non-ESBL) from 7/20 plants (4 downstream of hospitals). The third most common human lineage (ST95, 11 % blood cultures, 47 non-ESBL) was uncommon (0.5 %) in wastewater (2 non-ESBL from two different plants, one of which was downstream of a hospital). Similar numbers of ST131 and ST73 isolates were sequenced from plants in direct receipt of hospital waste (28 ST131 and 4 ST73) and those that were not (35 ST131 and 5 ST73).

Wastewater also contained the most common ST identified in livestock [ST10 (16 % livestock, 7 % wastewater, 2 % blood culture isolates)]. ST10 isolates were more likely to be isolated in rural (*n*=10/96, 10 %) than urban plants (17/292, 6 %), but this was not significant (*P*=0.16). In total, 26 and 21 STs were found in the wastewater collection that were specific to the human invasive or livestock collection, respectively, but comparison to data in Enterobase (https://enterobase.warwick.ac.uk/species/index/ecoli) revealed that most of these STs have been reported previously from both animal and human reservoirs.

**Fig. 4.**
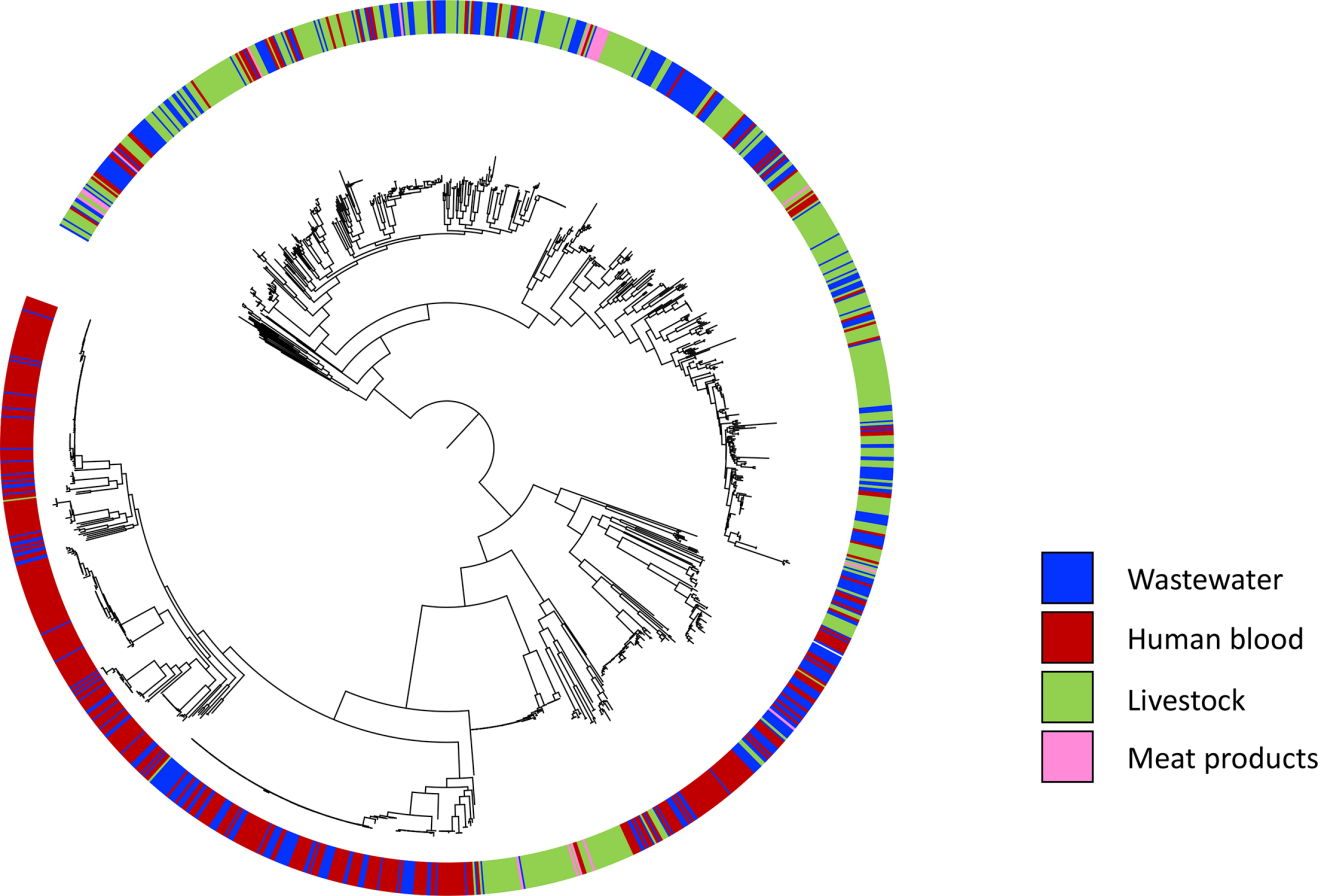
Relationship between *E. coli* from wastewater and isolates from humans, livestock and retail meat. Maximum-likelihood phylogenetic tree based on SNPs in the core genes for 386 wastewater isolates generated by this study, together with 437 human blood, 411 livestock and 20 retail meat isolates from the East of England region of the UK. The coloured ring shows the source of the isolates.

A phylogenetic tree of all 1254 isolates based on the core genome showed that wastewater isolates were distributed across the phylogeny and intermixed with isolates from humans and livestock (Fig. 4). Analysis of pairwise SNP differences in core genes revealed that all isolates within an arbitrary cut-off of 20 SNPs of wastewater isolates (*n*=24; ST44 *n*=1, ST69 *n*=1, ST131 *n*=20, ST404 *n*=1, ST648 *n*=1) were from bloodstream infections, with the exception of one wastewater isolate that was a minimum of 5 SNPs from multiple turkey isolates.

Mapping the sequence data to ST-specific reference genomes was performed for three STs to more accurately identify pairwise SNP differences between isolates from different reservoirs [ST10 and ST131 as described above, and ST117 which has been associated previously with turkeys and was positive for a distinct ESBL gene (*bla*_CTX-M-1_)]. ST10 wastewater isolates were distributed across the ST10 phylogeny and interspersed with both human and livestock isolates, although the majority (85 %) were most closely related to human isolates based on SNP difference (Fig. S3b). The smallest SNP difference for ST10 isolates was between two human isolates (60 SNPs). ST131 wastewater isolates were genetically intermixed with human clinical ST131 isolates, with a minimum of 21 SNPs between water/human isolates (Fig. S3a). The single ST117 wastewater isolate belonged to a *bla*_CTX-M-1_ poultry-associated lineage (Fig. S3c). This was 17 SNPs from a turkey isolate and carried *bla*_CTX-M-1_, suggesting that livestock lineages reach municipal wastewater.

### Antibiotic resistance

Mobile genes encoding resistance to colistin and to the carbapenem drugs were not detected in any wastewater isolate. Ten different *bla*_CTX-M_ genes were identified in wastewater isolates, the most common being *bla*_CTX-M-15_ (*n*=122, 64 %), *bla*_CTX-M-14_ (*n*=20, 10 %) and *bla*_CTX-M-27_ (*n*=20, 10 %). These three *bla*_CTX-M_ genes were also present in the bloodstream collection [*bla*_CTX-M-15_ (*n*=28, 49 %), *bla*_CTX-M-14_ (*n*=3, 6 %) and *bla*_CTX-M-27_ (*n*=5, 10 %)]. The most prevalent *bla*_CTX-M_ gene in livestock [*bla*_CTX-M-1_ 20 % (*n*=82) of livestock isolates, and 65 % (*n*=13) of meat isolates] was identified in wastewater isolates (*n*=12, 3%) but was rare in the human collection (*n*=1, 0.2 %). A further 79 antibiotic-resistance gene variants were identified in the 386 wastewater isolates, 21 of which were detected only in wastewater (Table S3). Eighteen gene variants identified in bloodstream isolates were absent from the wastewater collection: *aadA13, aadA24* and *aadA4* (aminoglycoside resistance); *bla*_CMY_, *bla*_SHV-48_ and four *bla*_TEM_ variants (β-lactam resistance); *fosA* (fosfomycin resistance); *mphB* and *ere_B* (macrolide resistance); *qnrB7* (quinolone resistance); ARR (rifampicin resistance); *tetA* (tetracycline resistance); *dfrA18*, *dfrA21* and *dfrB1* (trimethoprim resistance). However, all but *aadA13* (*n*=9), *bla*_SHV__-48_ (*n*=12), *mphB* (*n*=5) and *tetA* (*n*=2) were identified in only a single isolate (Table S3). The remaining 56 resistance gene variants identified in the bloodstream collection were also detected in wastewater.

We further explored whether the most prevalent *bla*_CTX-M_ genes (*bla*_CTX-M-15_, *bla*_CTX-M-1_, *bla*_CTX-M-27_, *bla*_CTX-M-14_ and *bla*_CTX-M-14b_) in isolates from wastewater, humans and livestock were carried by the same or different mobile genetic elements. Using this approach, we identified sharing of MGE clusters (Table S4) between wastewater and other reservoirs for *bla*_CTX-M-15_ (7 clusters containing a total of 35 wastewater, 17 human and 25 livestock isolates), *bla*_CTX-M-1_ (4 clusters: 7 wastewater, 17 livestock and 6 meat isolates), *bla*_CTX-M-27_ (3 clusters: 18 wastewater and 5 human isolates), *bla*_CTX-M-14_ (1 cluster; 2 wastewater, 1 human) and *bla*_CTX-M-14_b_ (1 cluster: 1 wastewater and 1 human isolate).

## Discussion

The most common *E. coli* lineage (ST131) associated with bloodstream infection in our region was isolated from all but two wastewater treatment plants and made up almost one fifth of the wastewater isolates sequenced. ST131 has also previously been identified in wastewater and rivers in Europe [17–20]. A phylogenetic analysis of ST131 based on core genome SNPs demonstrated that wastewater isolates were genetically intermixed with human clinical isolates, which is consistent with a human origin. However, the second and third most common human lineages (ST73 and ST95, which accounted for 16 and 11 % of blood culture isolates) were only identified from a minority of plants (7 and 2 plants, respectively) and a small proportion (2 and 0.5 %, respectively) of the isolates sequenced. This is likely due to our sampling strategy, since we selected equal numbers of non-ESBL and ESBL *E. coli* for sequencing (despite the lower abundance of ESBL *E. coli*) to enrich for genes encoding antibiotic resistance. This will skew the sequenced population in favour of ESBL *E. coli* lineages such as the C2 lineage in ST131 [13, 21]. ST73 and ST95 are reported to be predominantly ESBL-negative [13] and this, together with the high genetic diversity of non-ESBL *E. coli* in wastewater, could explain the low prevalence of these lineages in our collection. This demonstrates the importance of the sampling strategy if this tool is to be used for surveillance. Alternative explanations could be low prevalence of these lineages outside of the hospital setting or selection of ESBL lineages in wastewater. It has previously been suggested that selective pressure from antibiotics in sewage may provide an advantage for ESBL *E. coli* over non-ESBL *E. coli* clones in wastewater [7].

One explanation for this difference between lineages is that ST131 in wastewater is predominantly ESBL-positive (56/63, 89 %) and was selected from the background population using *Brilliance* ESBL agar. By contrast, ST73 and ST95 isolates were almost all ESBL-negative, and their representation in a given sample may have been masked by other lineages using agar that enriches for *E. coli* and coliforms alone. This possibility is further supported by the finding that the number of STs was greater in the non-ESBL *E. coli* population versus the ESBL *E. coli* population. We gave equal numerical weighting to non-ESBL and ESBL *E. coli* during selection of isolates for sequencing in order to enrich for genes encoding antibiotic resistance, but this skews the underlying population in favour of ESBL *E. coli* (which were less abundant than non-ESBL *E. coli*). These findings suggest that genomic surveillance for dominant human lineages that are antibiotic resistant is possible and aided by the use of selective media, but that this has more limited sensitivity for the detection of specific non-resistant lineages.

The inclusion of isolates from livestock at farms located in the same geographical region as the wastewater treatment plants allowed us to determine whether lineages from livestock were also represented in sewage. Wastewater contained the most common ST (ST10) identified in livestock, which was uncommon in the blood culture isolates. This suggests that livestock lineages may be represented in sewage. Furthermore, a single ST117 wastewater isolate belonged to a *bla*_CTX-M-1_ poultry-associated lineage and was highly related to a turkey isolate that also carried *bla*_CTX-M-1_. However, the low frequency of these relatedness events is consistent with farm-level management of waste from livestock.

Surveillance of *E. coli* genes encoding resistance to key antibiotic drug groups provided a wealth of information. Culture-based methods using a selective medium to enrich for ESBL-*E. coli* showed that this was almost ubiquitous in wastewater samples (39/40 from 20 plants), consistent with previous studies [10, 22]. There was no statistical difference in ESBL *E. coli* counts in wastewater treatment plants in direct receipt of waste from acute hospitals versus those that were not. Shotgun metagenomics have been used to investigate microbial populations, antimicrobial-resistance genes and mobile genetic elements in wastewater located upstream and downstream of a nursing home in Germany, which also reported no differences between upstream and downstream samples [23]. The dominant ESBL gene in clinical isolates (*bla*_CTX-M-15_) was detected in all 20 wastewater plants. Its ubiquitous presence indicates that monitoring of trends over time would require quantitative rather than qualitative monitoring, which is not well suited to individual colony sequencing. We also detected a wide range of other resistance genes to all of the major antibiotic groups. By contrast, we did not detect genes encoding resistance to the carbapenem drugs or colistin, which is consistent with the very low rate of isolation of *E. coli* that are resistant to these drugs in the East of England [2, 24]. Monitoring for these genes could provide early warning of their introduction and dissemination.

The finding that there was no statistical difference in the counts of *E. coli* and ESBL-*E. coli* in plants downstream of and unrelated to acute hospitals, combined with identification of similar STs between these plants, suggests that clinical lineages and ESBL-*E. coli* may be widespread in the community. Little is known about *E. coli* lineages and their resistance genes in the general population, but wastewater has been proposed as a surrogate for the human gut [25]. Surveillance of *E. coli* carriage in the community is important since there are ~40 000 *E*. *coli* bloodstream infections a year in England, and three-quarters of these develop before admission to hospital [26]. Whilst hospital-based surveillance using rectal screening has the potential to provide insights into nosocomial lineages, such screening is not universal and rectal swabs are relatively invasive. Our findings that the dominant *E. coli* lineages and resistance genes associated with bloodstream infections can be detected in wastewater suggests that this could serve as an accessible, non-invasive resource for surveillance of both the community and hospital reservoirs. However, further investigation is required to compare the utility of such pooled sampling versus direct sampling of individuals.

Our study also allowed us to determine the effect of the wastewater treatment process on *E. coli* counts in our region. All types of wastewater treatment process led to a reduction in *E. coli* counts, as described previously [7, 17]. Secondary and tertiary treatment led to similar levels of *E. coli* reduction overall, but terminal UV light treatment was the most effective. However, even the most stringent of wastewater treatments (tertiary treatment including UV light) did not eradicate ESBL-*E. coli* from the majority of wastewater effluent samples. Other studies have demonstrated contamination of surface waters downstream of untreated sewer overflows [27], and river sediments downstream of municipal wastewater plants [28]. The importance of this for clinical and animal health is not clearly understood.

A limitation of our study is that we only sampled plants in one defined geographical region in England and at one time point, and our findings may differ compared with plants in other regions and may change over time within each plant. In addition, we did not sample the community in this study, and the majority of our clinical isolates were taken at a different time-point to the wastewater isolates. Whilst this may affect the lineages detected due to shifts in the prevalence of lineages over time, it is unlikely to have affected our analysis of genetic relatedness between reservoirs since the mutation rate of *E. coli* is very low (approximately 1 SNP per genome per year) [29, 30].

In conclusion, our study suggests that sampling of sewage has the potential to provide regional information on bacterial lineages and resistant genes of critical importance, such as those encoding resistance to the carbapenem drugs and colistin, although the detection of non-ESBL *E. coli* lineages is more challenging. Further studies are required to evaluate whether longitudinal sampling can reliably track changes in lineages and resistance genes over time.

## Data Bibliography

1. Kallonen T, Brodrick HJ, Harris SR, Corander J, Brown NM, *et al*. European Nucleotide Archive, PRJEB4681 (2017).
  2. Ludden C, Raven KE, Jamrozy D, Gouliouris T, Blane B, *et al*. European Nucleotide Archive; PRJEB8774, PRJEB8776 (2018).

## Supplementary Data

Supplementary File 1

Supplementary File 2
