# Supplementary material for: Genomic surveillance of Escherichia coli in municipal wastewater treatment plants as an indicator of clinically relevant pathogens and their resistance genes
Source: Microb Genom. 2019 May 20;5(5):e000267. doi: 10.1099/mgen.0.000267 (PMC6562247; doi:10.1099/mgen.0.000267)
Supplement: Supplementary File 1 [file mgen-5-267-s001.pdf]

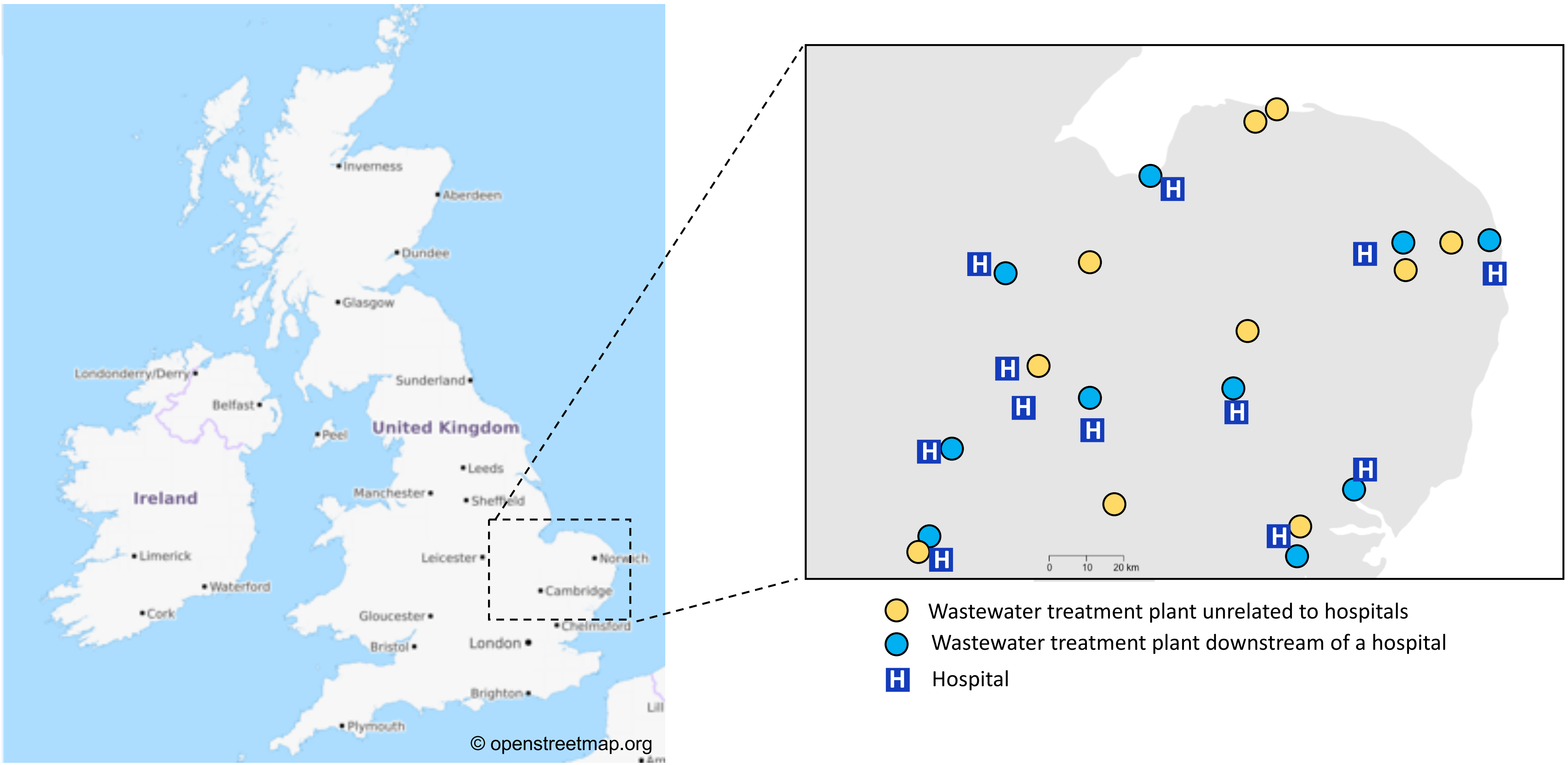

Figure S1. Map showing the United Kingdom (left hand side) and a close-up of the East of England region of the UK (right hand side) showing the location of the wastewater treatment plants sampled in the study, and the locations of hospitals in the East of England.

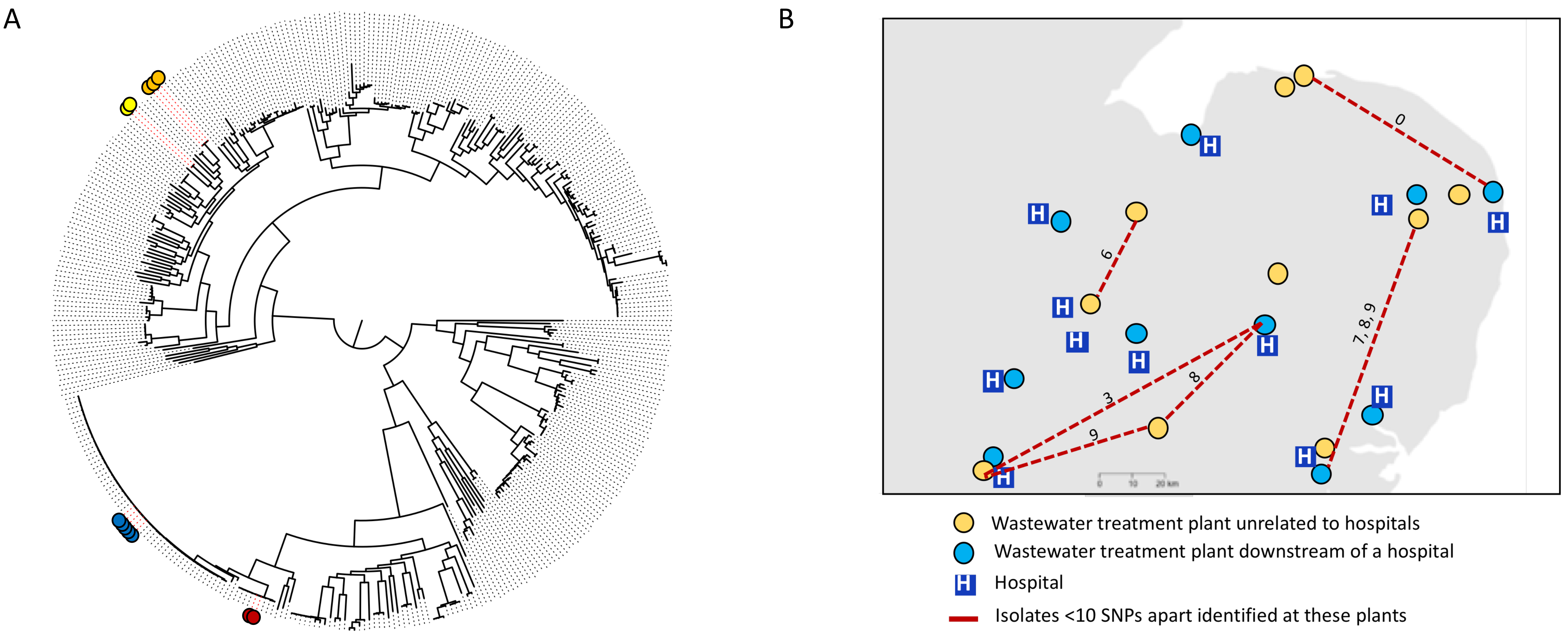

Figure S2. Genetically related isolates identified at different wastewater treatment plants. A: Phylogeny of the wastewater isolates based on SNPs in the core genes, showing clusters of isolates linked by less than 10 SNPs (coloured circles). B: Map showing the locations of wastewater treatment plants with red lines linking plants sharing isolates less than 10 SNPs apart. The numbers on the lines show the number of SNPs different between the isolates from the two plants.

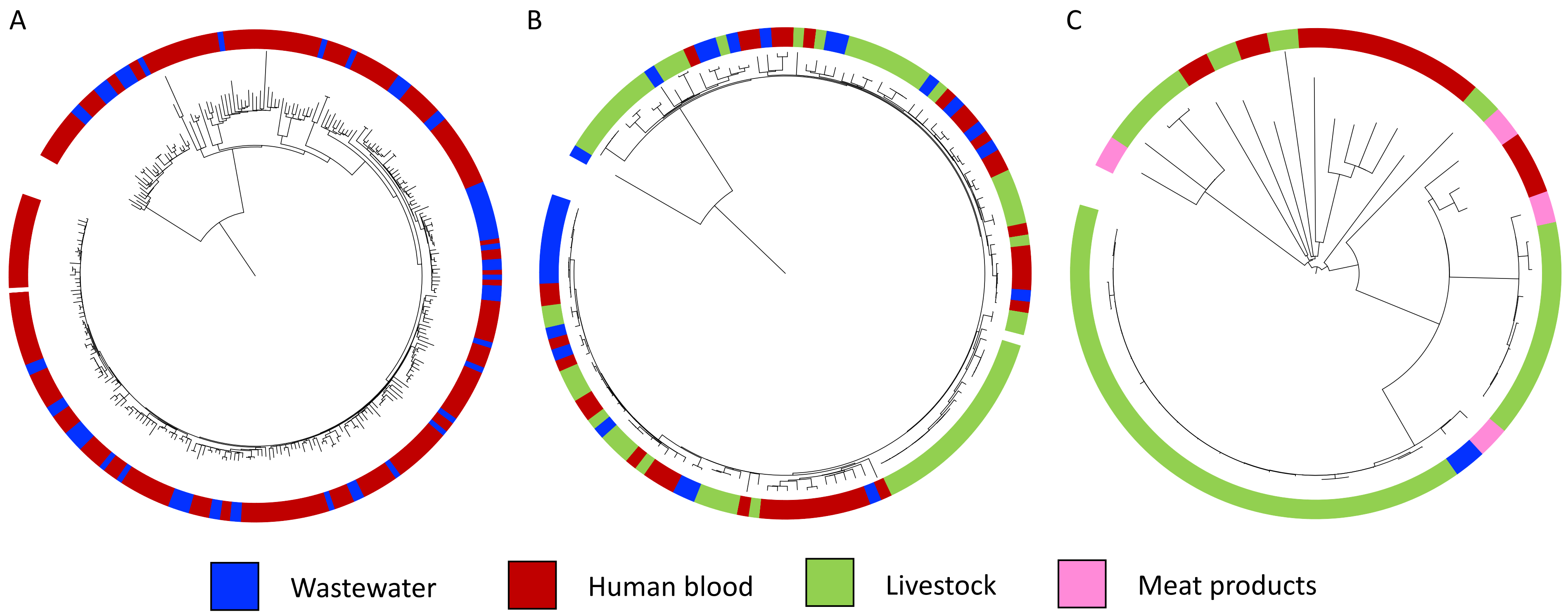

Figure S3. Relationship of wastewater isolates to other reservoirs for ST131, ST10 and ST117. Maximum likelihood phylogenetic trees of (A) ST131, (B) ST10 and (C) ST117 *E. coli* isolates based on mapping to a reference isolate. Outer ring shows the source of the isolates (blue = wastewater, green = livestock, red = human blood, pink = meat).
